# Supplementary material for: Efficacy of Unsupervised Self-Collected Mid-Turbinate FLOQSwabs for the Diagnosis of Coronavirus Disease 2019 (COVID-19)
Source: Viruses. 2021 Aug 22;13(8):1663. doi: 10.3390/v13081663 (PMC8402664; doi:10.3390/v13081663)
Supplement: Supplementary file 1 [file viruses-13-01663-s001.zip › viruses-1249897-supplementary.pdf]

Supplemental Table S1. Diagnostic sensitivity, specificity, positive and negative predictive values

	SC Swabs vs HCP Swabs	
	**Ratio %**	**95% Confidence Interval**
Sensitivity	94,23	84,05- 98,79
Specificity	95,65	78,05-99,89
PPV[a]	98,00	87,80-99,70
NPV[b]	88,00	70,90- 95,67
Positive Likelihood Ratio	21,67	3,18-147,55
Accuracy	94,67	86,90-98,53

[a]***PPV:*** Positive Predictive Value, [b]***NPV***:Negative Predictive Value

Supplemental Table S2. Results of qRT-PCR performed on SC swabs immediately placed in eNAT medium (t0) and SC swabs dry stored at room temperature for 6 days.

		SC swabs in eNAT		SC swabs dry	
**Sample ID**	**Target**	**Ct[a]**	**Result[b]**	**Ct[a]**	**Result[b]**
#A	MS2	27,9	Positive	28,1	Positive
	N-gene	34,1		33,9	
	ORF1ab	33,7		34	
	S-gene	37,4		37,1	
	B- globin	28,1	Valid	28,3	Valid
#B	MS2	27,9	Positive	28,2	Positive
	N-gene	34,1		34,3	
	ORF1ab	33,7		33,9	
	S-gene	37,4		37	
	B- globin	29,8	Valid	29,7	Valid
#C	MS2	26,4	Negative	27,1	Negative
	N-gene	Undetermined		Undetermined	
	ORF1ab	Undetermined		Undetermined	
	S-gene	Undetermined		Undetermined	
	B- globin	27,4	Valid	26,9	Valid
#D	MS2	24,7	Negative	24,9	Negative
	N-gene	37,5		37,8	
	ORF1ab	Undetermined		Undetermined	
	S-gene	Undetermined		Undetermined	
	B- globin	29,4	Valid	29,8	Valid
#E	MS2	24,7	Negative	24,1	Negative
	N-gene	37,5		37,3	
	ORF1ab	Undetermined		Undetermined	
	S-gene	Undetermined		Undetermined	
	B- globin	28,4	Valid	29,1	Valid

[a]Ct of Viral genes, MS2 control, and b-globin
[b]The test results

Supplemental Table S3. Diagnostic sensitivity, specificity, positive and negative predictive values

	SC Swabs vs HCP Swabs	
	**Ratio %**	**95% Confidence Interval**
Sensitivity	94,23	84,05- 98,79
Specificity	95,65	78,05-99,89
PPV[a]	98,00	87,80-99,70
NPV[b]	88,00	70,90- 95,67
Positive Likelihood Ratio	21,67	3,18-147,55
Accuracy	94,67	86,90-98,53

[a]***PPV:*** Positive Predictive Value, [b]***NPV***:Negative Predictive Value

Supplemental Table S4. Results of the *in vitro* stability test. B-globin was reported separately.

**Sample**	**Target**	**Ct**	**Sample**	**Target**	**Ct**
NEG 0h	MS2	22,37	POS 0h	MS2	23,68
NEG 0h	N-gene	Undetermined	POS 0h	N-gene	23,83
NEG 0h	ORF1ab	Undetermined	POS 0h	ORF1ab	23,42
NEG 0h	S-gene	Undetermined	POS 0h	S-gene	22,64
NEG 48h RT	MS2	22,99	POS 48h RT	MS2	24,46
NEG 48h RT	N-gene	Undetermined	POS 48h RT	N-gene	23,90
NEG 48h RT	ORF1ab	Undetermined	POS 48h RT	ORF1ab	22,16
NEG 48h RT	S-gene	Undetermined	POS 48h RT	S-gene	23,29
NEG 48h 4°C	MS2	22,52	POS 48h 4°C	MS2	24,19
NEG 48h 4°C	N-gene	Undetermined	POS 48h 4°C	N-gene	22,39
NEG 48h 4°C	ORF1ab	Undetermined	POS 48h 4°C	ORF1ab	23,15
NEG 48h 4°C	S-gene	Undetermined	POS 48h 4°C	S-gene	23,27
NEG 72h RT	MS2	23,79	POS 72h RT	MS2	22,95
NEG 72h RT	N-gene	Undetermined	POS 72h RT	N-gene	23,75
NEG 72h RT	ORF1ab	Undetermined	POS 72h RT	ORF1ab	23,32
NEG 72h RT	S-gene	Undetermined	POS 72h RT	S-gene	22,99
NEG 72h 4°C	MS2	22,79	POS 72h 4°C	MS2	23,29
NEG 72h 4°C	N-gene	Undetermined	POS 72h 4°C	N-gene	23,03
NEG 72h 4°C	ORF1ab	Undetermined	POS 72h 4°C	ORF1ab	22,87

**Sample**	**Target**	**Ct**	**Sample**	**Target**	**Ct**
NEG 72h 4°C	S-gene	Undetermined	POS 72h 4°C	S-gene	23,39
NEG 6d RT	MS2	24,29	POS 6d RT	MS2	23,71
NEG 6d RT	N-gene	38,66	POS 6d RT	N-gene	22,92
NEG 6d RT	ORF1ab	Undetermined	POS 6d RT	ORF1ab	22,08
NEG 6d RT	S-gene	Undetermined	POS 6d RT	S-gene	23,08
NEG 6d 4°C	MS2	23,60	POS 6d 4°C	MS2	24,15
NEG 6d 4°C	N-gene	Undetermined	POS 6d 4°C	N-gene	23,41
NEG 6d 4°C	ORF1ab	Undetermined	POS 6d 4°C	ORF1ab	23,20
NEG 6d 4°C	S-gene	Undetermined	POS 6d 4°C	S-gene	23,62
NEG 0h	B GLOBIN	30,71	POS 0h	B GLOBIN	31,97
NEG 48h RT	B GLOBIN	31,22	POS 48h RT	B GLOBIN	32,5
NEG 48h 4°C	B GLOBIN	31,35	POS 48h 4°C	B GLOBIN	32,7
NEG 72h RT	B GLOBIN	30,86	POS 72h RT	B GLOBIN	32,02
NEG 72h 4°C	B GLOBIN	31,36	POS 72h 4°C	B GLOBIN	32,84
NEG 6d RT	B GLOBIN	31,78	POS 6d RT	B GLOBIN	32,42
NEG 6d 4°C	B GLOBIN	30,75	POS 6d 4°C	B GLOBIN	32,6
